# From Theory to Competence: A Conceptual Review of Humanized Care in Community Nursing in the Ecuadorian Context

**DOI:** 10.3390/healthcare14193257

**Published:** 2026-10-01

**Authors:** María Ximena Tapia-Paguay, Eva Consuelo López-Aguilar, Amparo Paola Tito-Pineda, Eloy Fernández-Cusimamani, Viviana Margarita Espinel-Jara

**Affiliations:** 1 Department of Nursing, Faculty of Health Sciences, Universidad Tecnica del Norte, Avda. 17 de Julio 5-21, Ibarra 100105, Ecuador; mxtapia@utn.edu.ec (M.X.T.-P.); eclopez@utn.edu.ec (E.C.L.-A.); aptito@utn.edu.ec (A.P.T.-P.); vmespinel@utn.edu.ec (V.M.E.-J.); 2 Department of Crop Sciences and Agroforestry, Faculty of Tropical AgriSciences, Czech University of Life Sciences Prague, Kamýcká 129, 165 00 Prague, Czech Republic

**Keywords:** Dorothea Orem, Ecuador, Jean Watson, Katharine Kolcaba, nursing theory integration, professional competence, RCC framework

## Abstract

Community nursing in Latin America requires a shift from control-oriented biomedical models toward relational and culturally responsive approaches capable of addressing persistent structural inequalities and intercultural complexity. This review develops the Relate–Capacity–Comfort (RCC) framework. This integrated model synthesizes three foundational nursing theories: Jean Watson’s Theory of Human Caring, Dorothea Orem’s Self-Care Deficit Nursing Theory, and Katharine Kolcaba’s Comfort Theory. The framework conceptualizes community nursing as a sequential process comprising relational engagement (Relate), strengthening of self-care capacity (Capacity), and achievement of experienced comfort (Comfort) as an outcome. Grounded in the Ecuadorian primary healthcare context where biomedical and Indigenous systems intersect within conditions of ongoing socioeconomic disparity, this review translates theoretical constructs into operational professional competence. Addressing a gap in the literature, the RCC framework integrates the relational engagement, self-care capacity, and experienced comfort into a coherent, practice-oriented structure aligned with the realities of community-based nursing. Evidence from Ecuadorian contexts indicates that humanized, integrated approaches are associated with increased trust, improved treatment adherence, enhanced patient satisfaction, and progress toward health equity, particularly in resource-limited rural settings. By providing a concise and contextually grounded model, the RCC framework supports the transition from theoretical knowledge to embodied practice. This review positions the RCC framework as a practical, scalable contribution to community nursing, demonstrating that theory-informed, culturally responsive care is essential to advancing equitable, person-centered health systems in Latin America.

## 1. Introduction

Community nursing calls for a fundamental shift away from hospital-centered, control-oriented biomedical models toward relational, context-sensitive approaches grounded in human connection. Rather than prioritizing standardized interventions, contemporary community practice emphasizes authentic engagement with individuals, families, and communities. This transformation is particularly evident in rural Ecuadorian settings, where nurses function not merely as health promoters but as enabling agents embedded within the social and territorial fabric of the populations they serve. Through sustained home visits, intercultural dialogue, and participatory action, community nurses adapt care practices to local realities shaped by geographic isolation, limited resources, and fragmented health systems [1].

In Ecuador, national healthcare reforms implemented between 2007 and 2017 significantly expanded primary care coverage by establishing Basic Health Care Teams (Equipos Básicos de Atención en Salud, EBAS) and introducing free access to healthcare services. These reforms contributed to a reduction in unmet healthcare needs, which declined from 27% in 2006 to 18% in 2014. However, despite these advances, persistent socioeconomic inequalities continue to limit equitable access to care. Rural populations, Indigenous communities, and individuals of lower socioeconomic status remain disproportionately affected by structural barriers, highlighting the limitations of system-level improvements in addressing deeply rooted disparities [2].

Within this context, humanized, community-based nursing emerges as a critical response to inequity. Humanized care extends beyond clinical tasks to encompass holistic, person-centered practices that affirm dignity, emotional well-being, and social connectedness. This approach is especially relevant for vulnerable populations, such as older adults in low-resource environments, where loneliness and marginalization compound health risks [3,4]. Phenomenological studies further illuminate humanized care as an ethical and relational commitment characterized by empathy, active listening, and authentic presence, positioning the nurse-patient relationship as central to care delivery [5].

Despite the growing recognition of these relational engagements, a critical gap persists in both theory and practice. Existing nursing frameworks are often applied in isolation and lack integration into a coherent, practice-oriented model that reflects the dynamic progression of care in community settings. There is limited guidance on systematically integrating relational engagement, self-care capacity, and experienced comfort within a unified framework. This gap is particularly significant in intercultural and resource-constrained contexts, where adaptability and continuity of care are essential.

Although humanized care is commonly associated with dignity, empathy, therapeutic relationships, participation, and experienced comfort [3,4,5], these elements are rarely organized into a theoretically connected process of community nursing practice. The RCC Framework, therefore, draws on Watson, Orem, and Kolcaba because their theories provide three distinct and complementary functions required to conceptualize humanized care as relational engagement, development of patient agency, and experienced well-being. Watson’s Theory of Human Caring establishes the relational engagement through authentic presence, empathy, and human connection [6,7,8,9,10,11,12], Orem’s Self-Care Deficit Nursing Theory provides the agency mechanism through which nursing strengthens individuals’ capacity for self-care and participation [13,14,15,16,17], and Kolcaba’s Comfort Theory provides the experiential comfort framework by defining well-being across physical, psychospiritual, sociocultural, and environmental dimensions [18,19,20,21,22,23]. Self-care capacity is consequently positioned not as a defining attribute of humanized care, but as the mechanism linking relational care with patient participation and well-being. This integration addresses a gap in the existing humanized-care literature, which has predominantly examined relational attributes, competencies, or interventions in isolation, with less attention to their integration within a community-oriented, intercultural framework [4].

This review seeks to address this limitation by proposing an integrated framework that synthesizes three foundational nursing theories: the Theory of Human Caring, the Self-Care Deficit Nursing Theory, and Comfort Theory. The resulting Relate-Capacity-Comfort (RCC) Framework conceptualizes community nursing as an interconnected and context-sensitive process. It begins with the establishment of a meaningful therapeutic relationship engagement (Relate), progresses through the strengthening of individuals’ self-care capacities (Capacity), and culminates in the experience of holistic comfort (Comfort) as a measurable outcome.

The RCC Framework is particularly relevant in the Ecuadorian context, where healthcare policy and nursing education increasingly prioritize humanization, intercultural competence, and primary healthcare approaches [24,25]. By translating abstract theoretical constructs into a structured and practical model, this review aims to support community nurses in navigating complex care environments with clarity and intentionality.

Ultimately, this work positions theory not as an abstract body of knowledge but as a practical tool for guiding everyday clinical decision-making. By integrating relational engagement, developmental, and outcome-oriented dimensions of care, the proposed framework offers a pathway to strengthen trust, enhance adherence, promote equity, and improve overall well-being in underserved communities. Community nursing, in this sense, is not defined by control, but by connection, anchored in dignity, respect, and the lived realities of the populations it serves.

## 2. Literature Identification and Conceptual Synthesis

### 2.1. Literature Identification

This conceptual review was developed through a targeted identification and critical synthesis of literature relevant to humanized care, community nursing, nursing theory, professional competence, and the Ecuadorian and broader Latin American healthcare context. Relevant literature was identified through searches of major scholarly databases using combinations of keywords related to humanized care, community nursing, Watson’s Theory of Human Caring, Orem’s Self-Care Deficit Nursing Theory, Kolcaba’s Comfort Theory, self-care capacity, patient participation, intercultural nursing, and Ecuadorian and Latin American primary healthcare. Additional relevant publications were identified through reference-list examination of key articles and theoretical sources. Literature was selected for its conceptual relevance to the review’s objectives and its contribution to understanding the relational, self-care, experiential, and contextual dimensions of community nursing.

The purpose of the literature identification process was to inform conceptual integration and framework development rather than to provide an exhaustive systematic review of the available evidence. Accordingly, the review did not employ a formal systematic screening or meta-analytic procedure. The identified literature was critically interpreted and synthesized to establish the conceptual relationships among relational engagement, self-care capacity, and experienced comfort, which subsequently informed the development of the Relate-Capacity-Comfort (RCC) Framework.

### 2.2. Selection of Theoretical Foundations

The theoretical foundations of the RCC Framework were selected through a concept-oriented appraisal of nursing theories relevant to humanized, person-centered, and community-based care. Candidate theories were considered according to four predefined conceptual criteria: (i) direct relevance to humanized and person-centered nursing care; (ii) ability to explain a distinct functional dimension of the proposed care process; (iii) applicability to community-based nursing and patient participation; and (iv) potential for integration with other theoretical perspectives without substantial conceptual redundancy.

Based on these criteria, Watson’s Theory of Human Caring, Orem’s Self-Care Deficit Nursing Theory, and Kolcaba’s Comfort Theory were selected because each contributes a distinct but complementary function to the proposed framework. Watson provides the relational foundation through authentic presence, empathy, and human connection, corresponding to the Relate dimension. Orem provides the agency and self-care dimension through the development and exercise of individual self-care abilities, corresponding to Capacity. Kolcaba provides the experiential outcome dimension by conceptualizing comfort across physical, psychospiritual, sociocultural, and environmental contexts, corresponding to Comfort. Their combined application therefore provides a coherent conceptual progression from relational engagement, through development of self-care capacity, to experienced comfort.

Other relevant nursing theories were also considered in relation to the conceptual scope of the framework, but were not incorporated as additional core components when their principal theoretical contribution did not correspond to a distinct functional stage within the proposed RCC sequence. Leininger’s Culture Care Diversity and Universality Theory was recognized as highly relevant to culturally congruent and intercultural nursing, particularly within the Ecuadorian context. However, the purpose of the RCC Framework was not to develop a culture-care model. Instead, intercultural responsiveness was conceptualized as a cross-cutting contextual principle influencing all three RCC dimensions. Thus, cultural and intercultural considerations are integrated across Relate, Capacity, and Comfort rather than represented as a separate sequential component. This approach enabled the framework to maintain a parsimonious three-domain structure while acknowledging the cultural context in which community nursing takes place.

## 3. Conceptual Core and Theoretical Foundation of Humanized Care in Community Nursing

Humanized care in community nursing can be conceptualized through three interrelated dimensions: relational engagement, self-care capacity, and experienced comfort [3,5]. Together, these dimensions present a coherent foundation for understanding how care is represented in real-world community contexts, particularly within resource-constrained and intercultural environments [1].

The relational engagement centers on the quality of interactions among nurses, individuals, their families, and their communities. It emphasizes authentic presence, empathy, and the development of transpersonal connections that foster trust, respect, and shared meaning. In community settings, these relationships are not incidental but foundational, shaping both access to care and its effectiveness. Phenomenological accounts describe humanized care as a deeply relational and ethical engagement, characterized by genuine presence and the co-creation of supportive spaces grounded in mutual recognition and dignity [5].

The self-care dimension highlights individuals’ role as active agents in their own health. Drawing on principles of self-care, this dimension emphasizes strengthening patients’ capacity to manage their health with appropriate support [13,14]. Rather than positioning individuals as passive recipients of care, community nursing reframes them as participants with the potential for self-determination. Evidence from interventions grounded in self-care theory demonstrates significant improvements in self-efficacy, quality of life, and functional independence, particularly among populations with chronic conditions and those in vulnerable settings [15,16,17]. In practice, nurses assess self-care capacity within the realities of community life and design context-sensitive strategies that align individual empowerment with broader public health goals [3].

Importantly, humanized care challenges institutional biases by reframing patients as active participants rather than passive consumers of healthcare services [3,5]. It extends beyond technical tasks, such as medication administration or physiological monitoring, to include emotional support, cultural respect, and ethical responsibility [1]. This expanded scope is particularly critical in Ecuadorian contexts, where care is often delivered amid structural adversity, geographic isolation, and fragmented health systems. In such settings, nurses frequently serve as the primary link between communities and formal healthcare systems, underscoring the need for relational and context-sensitive approaches [2].

Cultural competence is a central element of this model [26]. In rural Latin America, effective community nursing requires an intercultural perspective that values traditional knowledge [26] and local health practices rather than imposing biomedical dominance. Systematic evidence indicates that culturally congruent care enhances engagement, supports prevention, and mitigates the effects of structural inequities that contribute to elevated mortality and unmet health needs in underserved populations [1]. Community health workers further strengthen this interface by bridging formal health systems with local realities, although power imbalances may at times constrain these relationships [27].

Trust emerges as a critical mechanism that binds relational engagement, self-care capacity, and experiential comfort. In rural and marginalized communities, trust is cultivated through sustained presence, home visits, and active participation in everyday life. It facilitates adherence, community engagement, and more equitable outcomes [28]. However, trust remains fragile in contexts shaped by historical inequities and systemic limitations, as evidenced by public health crises such as the COVID-19 pandemic, during which disparities in humanized care experiences between urban and rural populations became more pronounced [2].

**Figure 1:**
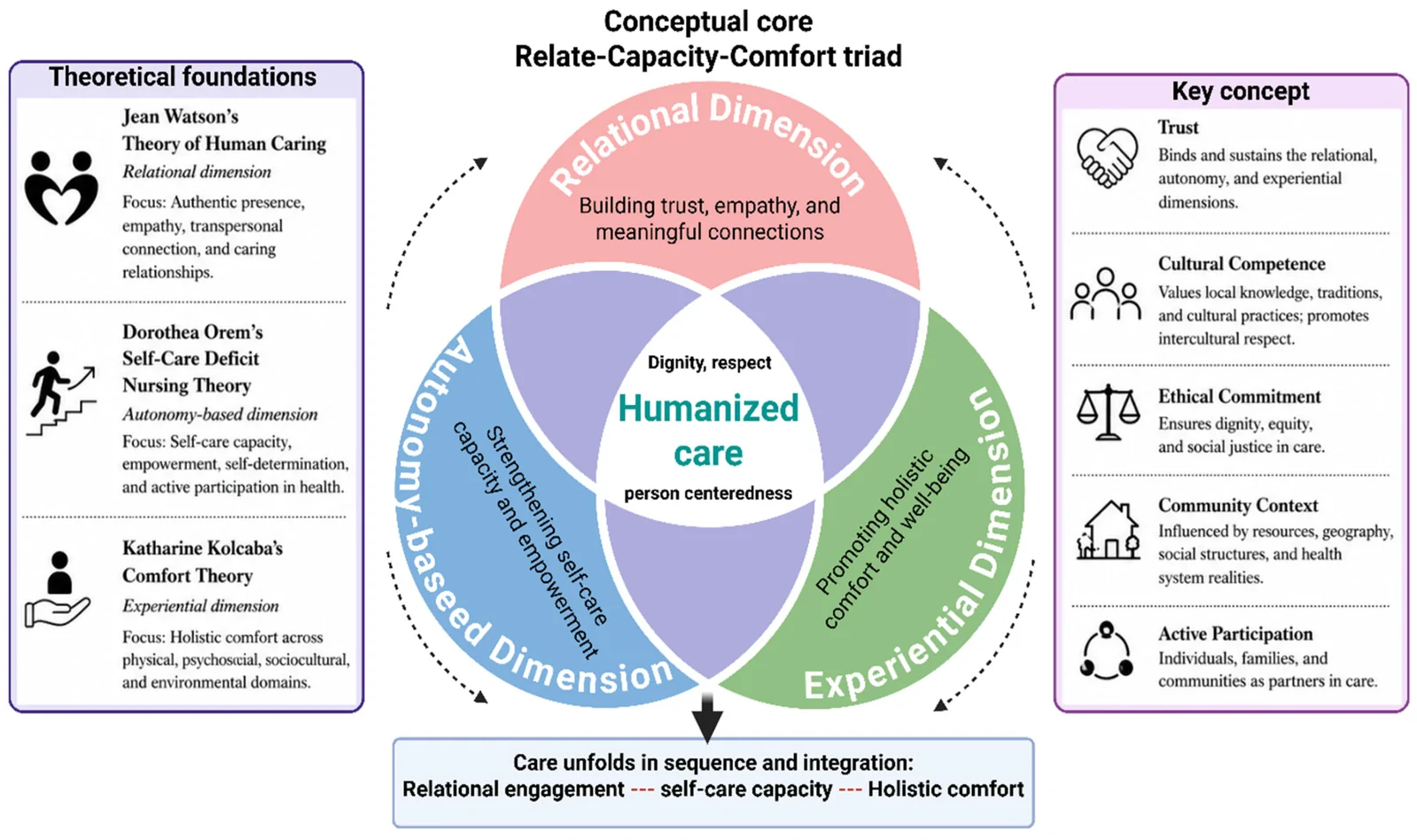
Conceptual foundation of humanized care in community nursing, which is grounded in the integration of relational engagement, self-care capacity, and the pursuit of holistic comfort within intercultural and resource-constrained community contexts.

The proposed RCC framework is grounded in this conceptual triad (Figure 1). It integrates Watson’s relational engagement, Orem’s focus on autonomy and self-care, and Kolcaba’s orientation toward comfort as an outcome. This synthesis enables a structured yet flexible approach to community nursing, in which care begins with relational engagement, progresses through the development of self-care capacity, and culminates in experienced comfort. As such, the framework offers a practical lens for addressing the complexities of intercultural healthcare delivery in Latin America, particularly in contexts where equity remains an ongoing challenge [24,25,29,30].

Experiential comfort addresses how individuals perceive comfort and well-being across physical, emotional, sociocultural, and environmental domains. This dimension recognizes that health outcomes extend beyond clinical indicators to include subjective experiences of dignity, relief, and social connectedness. In this sense, comfort becomes not merely an outcome but an integral component of care, reflecting the extent to which interventions resonate with individuals’ and communities’ lived experiences.

Together, these three dimensions form a conceptual triad that aligns closely with three foundational nursing theories: Jean Watson’s Theory of Human Caring, Dorothea Orem’s Self-Care Deficit Nursing Theory, and Katharine Kolcaba’s Comfort Theory. When integrated, these theories move beyond abstract constructs and provide the basis for a sequential, practice-oriented framework in which care unfolds in layers of relational engagement, the empowerment of self-care capacity, and the attainment of experience comfort.

Jean Watson’s Theory of Human Caring provides the relational and ethical basis by positioning caring as a transpersonal, moral practice rooted in authentic human connection [6]. Evolving from the original “carative factors” to the Caritas Processes, the theory emphasizes compassion, intentional presence, and the co-construction of meaning within nurse-patient relationships [7,8]. Empathy, mindfulness, and spiritual sensitivity are central, reframing care as an engaged, relational act rather than a series of technical tasks [9,10]. Also, reports from community and phenomenological studies show relational practices foster trust, respect, and therapeutic engagement, particularly in home-based care and sustained family interactions [5,11]. In Latin American contexts marked by intercultural diversity and structural inequities, this relational orientation aligns closely with the need for culturally responsive, dignity-centered care [12], establishing connection as the essential entry point to effective practice [1,24,25].

Building on this relational foundation, Dorothea Orem’s Self-Care Deficit Nursing Theory introduces an autonomy-centered perspective that defines nursing as the facilitation of individuals’ and communities’ self-care capacities [15]. Orem conceptualizes self-care as deliberate actions undertaken to maintain health, with nursing intervention required when individuals are underdeveloped due to a lack of knowledge, skills, or resources [14,16]. The theory’s supportive-educative orientation is particularly relevant in community settings, where nurses assess self-care capacities and design interventions that strengthen, rather than replace, patient agency [13,17]. Empirical evidence demonstrates that Orem-based approaches improve self-efficacy, quality of life, and preventive health behaviors across both chronic and infectious conditions, particularly among underserved populations [16]. In resource-limited Ecuadorian contexts, this framework supports community-based education, family engagement, and the integration of local knowledge systems, positioning individuals as active participants in their own health while promoting sustainability and reducing dependency [1,3].

Katharine Kolcaba’s Comfort Theory complements these perspectives by defining comfort as a central, measurable outcome of nursing care encompassing physical, psychospiritual, sociocultural, and environmental dimensions [18,19,20]. By prioritizing patient experience alongside clinical indicators, the theory shifts evaluation toward the lived realities of care [21]. In community nursing, this translates into addressing emotional distress, improving home environments, and responding to social determinants that shape well-being, particularly in contexts affected by poverty and geographic isolation [3,22]. Evidence indicates that comfort-oriented care is associated with improved satisfaction, adherence, and perceived quality of care, reinforcing its relevance as a key outcome in humanized practice [23].

Taken together, these theories provide a coherent foundation for humanized community nursing, but their integration also requires critical consideration of their limitations and contextual tensions. Watson’s emphasis on authentic relational presence may be difficult to sustain where nurses face high workloads, limited time, and fragmented services. Orem’s autonomy-oriented approach may be constrained when poverty, low health literacy, limited resources, or family dependency restrict individuals’ capacity for self-care, and Kolcaba’s emphasis on comfort, although valuable for capturing lived experience, may be challenging to operationalize consistently across culturally diverse communities. Moreover, evidence supporting humanized and theory-informed interventions is not uniformly consistent, with improvements in self-efficacy, satisfaction, or engagement sometimes varying according to population, intervention intensity, and health-system context. These tensions suggest that the three theories should not be applied as universal or sequential prescriptions, but adapted to local social, cultural, and structural conditions. Implementation is particularly challenging in resource-constrained and intercultural settings. Geographic isolation, workforce shortages, inadequate infrastructure, fragmented referral pathways, and competing clinical priorities can limit continuity of relational care and sustained capacity-building. Intercultural practice adds further complexity because traditional health beliefs, language differences, gender roles, and community power structures may conflict with biomedical expectations or institutional models of care. Although community health workers and local knowledge can facilitate cultural bridging, unequal power relations may also reproduce rather than reduce inequities if community participation is tokenistic. Therefore, the RCC framework should be understood as a context-sensitive guide rather than a fixed model. Its effectiveness depends on nurses’ cultural humility, institutional support, community participation, and the availability of resources that enable relational engagement, self-care development, and meaningful comfort outcomes. This critical positioning strengthens the framework by acknowledging that humanized care is shaped not only by nursing theory and professional intent but also by the structural and sociocultural conditions in which care is delivered (Table 1).

## 4. Theory to Professional Competence: Proposed Integrated RCC Framework Model

The relevance of nursing theory ultimately depends on its capacity to inform and transform practice. In community nursing, particularly in Ecuadorian contexts, abstract theoretical knowledge is insufficient unless it is translated into competencies that address the realities of home-based care, intercultural dynamics, and resource constraints [6]. Although the Theory of Human Caring, the Self-Care Deficit Nursing Theory, and Comfort Theory are well established in the literature, they are most often addressed independently, with limited attention to their integration within community-based practice. This fragmentation creates a persistent gap. Nurses possess conceptual knowledge but lack structured guidance for its application in settings shaped by structural inequality, geographic isolation, and sociocultural diversity [1,9,23].

Within this context, translating theory into professional competence becomes both a practical necessity and an ethical imperative [31]. Humanized care in community nursing requires more than executing technical tasks; it involves integrating relational, ethical, and cultural dimensions into everyday practice [31]. Evidence from rural Latin America consistently shows that effective care emerges when nurses combine clinical expertise with emotional intelligence and cultural adaptability, enabling them to engage meaningfully with individuals and communities [1]. Phenomenological research further underscores that nurses who move beyond procedural roles toward relational and value-driven practice not only enhance patient outcomes but also experience greater professional fulfillment [5,11]. However, in the absence of an integrated and practice-oriented framework, such translation remains uneven and highly dependent on individual initiative, particularly in contexts such as Ecuador, where intercultural health systems are still evolving [24,25,29].

Professional competence in humanized community nursing can thus be understood as a multidimensional construct that combines clinical knowledge with relational capacity, cultural competence, and ethical responsiveness. It requires nurses to engage patients as active participants in their own care, navigate diverse belief systems and local knowledge, and collaborate effectively with families and community networks [1,32]. In many Latin American settings, nurses also function as key intermediaries between formal health systems and underserved populations, reinforcing the need for competencies that extend beyond institutional models of care [27]. Through sustained engagement, including home visits and community-based interventions, care shifts from an individual-centered approach to a collaborative, contextually embedded process that supports long-term health outcomes [33,34].

**Figure 2:**
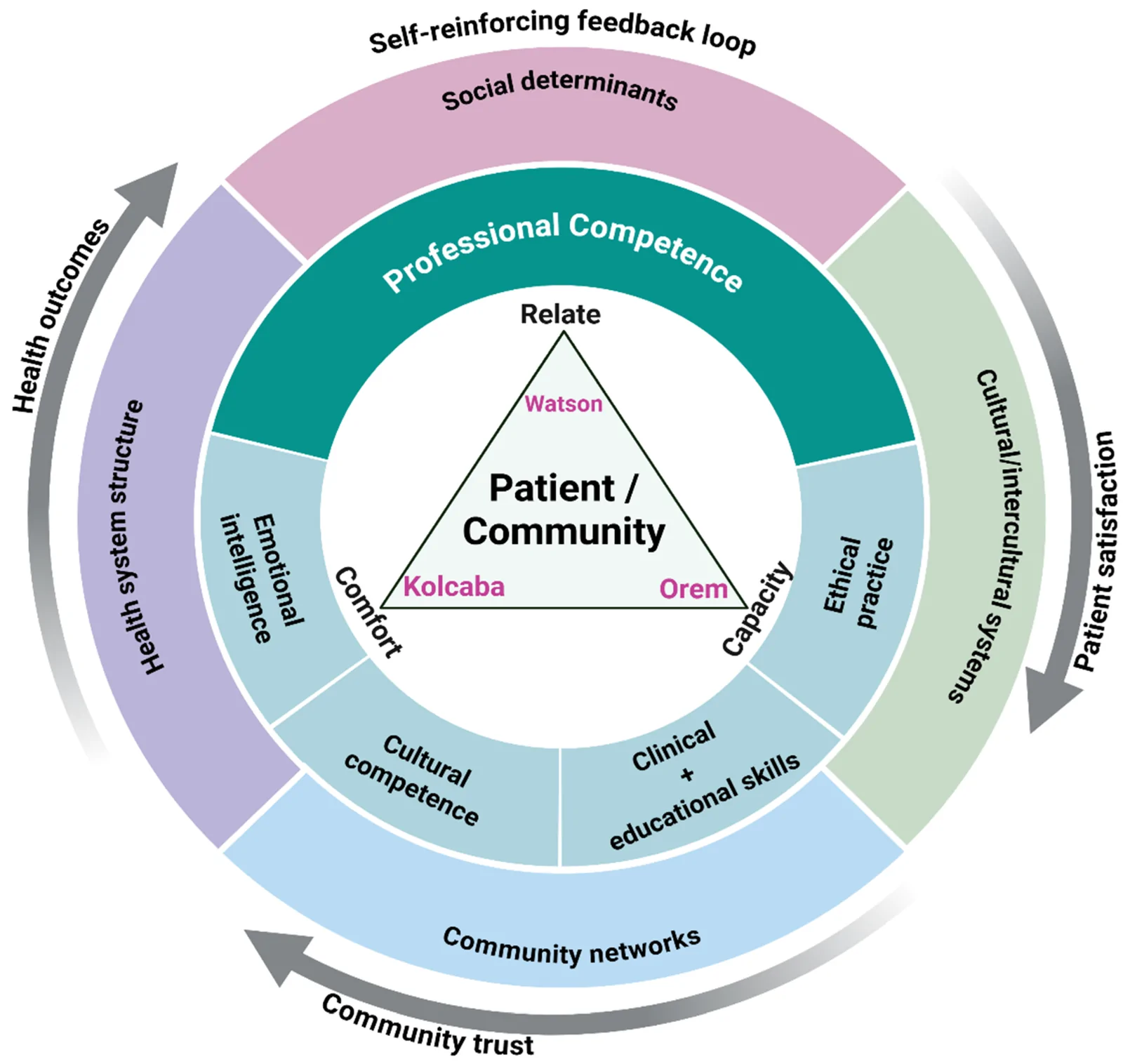
Conceptual ecosystem of humanized community nursing. This figure presents a conceptual framework synthesizing the literature reviewed in this article. It illustrates the proposed interrelationships among relational engagement (Relate), self-care development (Capacity), and experienced comfort outcomes (Comfort), supported by professional nursing competencies and influenced by sociocultural and structural contexts in Ecuador. The connecting pathways and feedback loops represent proposed conceptual relationships through which improved health outcomes, patient satisfaction, and community trust may influence subsequent care interactions.

In response to these demands, this review proposes the RCC framework as an integrated model for operationalizing theory into practice. The framework synthesizes key elements of Watson’s relational approach, Orem’s emphasis on self-care and autonomy, and Kolcaba’s focus on comfort and well-being into a coherent, sequential structure that reflects how care is enacted in community settings (Figure 2).

The Relate component establishes the foundation of care through trust-building and authentic human connection. Grounded in the principles of human caring, it emphasizes presence, empathy, and the recognition of emotional and spiritual dimensions of health [1]. In practice, this is achieved through sustained interaction, particularly in home and community environments, where trust becomes a prerequisite for engagement and acceptance of care. This relational grounding is especially critical in rural and marginalized settings, where historical inequities often shape perceptions of healthcare [29].

Building on this foundation, the Capacity component focuses on strengthening individuals’ ability to manage their own health. Drawing on self-care theory, it involves assessing capabilities, providing tailored education, and supporting autonomy through empowerment-oriented interventions [13,15]. Evidence demonstrates that such approaches improve self-efficacy, health behaviors, and quality of life, particularly among populations experiencing chronic illness and social vulnerability [14,16,17,32]. In resource-limited contexts, this emphasis on capacity-building is essential for sustainability, as it reinforces both individual independence and community resilience [1,3].

The final component, Comfort, addresses achieving experienced comfort as the outcome of care. Based on Comfort Theory, this dimension encompasses physical, psychospiritual, sociocultural, and environmental aspects of health [19,20]. It shifts the evaluation of care beyond clinical indicators to include individuals’ lived experiences of relief, dignity, and overall well-being [18]. Nurses contribute to this outcome by creating supportive environments, addressing social determinants, and aligning care with cultural and contextual realities [21,35]. This perspective is particularly significant for vulnerable populations, where perceived comfort directly influences adherence, satisfaction, and sustained engagement with care [3,36].

A central contribution of the RCC framework lies in its sequential and practice-aligned structure. By organizing care into the progression of relate, capacity, and comfort, the model reflects the natural flow of community nursing, where trust must be established before education is effective, and empowerment must be achieved before long-term well-being can be sustained [6]. This sequencing provides a clear, adaptable pathway for nurses, enabling them to navigate complex, unpredictable care environments while maintaining coherence in practice. Unlike approaches that apply theories in isolation, the RCC framework integrates them into a unified model that is both conceptually grounded and operationally relevant [15,20].

Importantly, the framework is closely aligned with the priorities of Latin American health systems, particularly in Ecuador, where interculturality, primary healthcare, and humanization of care are central yet still developing objectives. By embedding relational engagement, autonomy promotion, and experiential comfort into a single model, the RCC framework expands the notion of professional competence beyond technical proficiency [24]. It supports the development of practitioners who are not only clinically skilled but also culturally responsive, ethically grounded, and capable of addressing the broader determinants of health [25,30].

In this way, the RCC framework bridges the longstanding divide between theory and practice. It positions nursing theory as a functional tool for guiding action and decision-making in complex community contexts. More broadly, it reinforces humanized care as a core professional standard—one that is essential for addressing structural inequities and advancing health equity across Latin America.

## 5. Regional Application: Perspective and the Case of Ecuador

Humanized care in Latin America is closely linked to the pursuit of social justice and health equity. Community nurses serve as essential mediators between formal health systems and marginalized populations in contexts characterized by geographic isolation, persistent socioeconomic inequalities, and workforce shortages [1,22,37]. Their role extends beyond the delivery of standardized biomedical interventions to facilitate community participation, strengthen local engagement, and adapt care to lived realities. In this sense, humanized care is not an auxiliary feature of healthcare delivery but a necessary response to structural inequities that continue to shape access, quality, and outcomes across the region.

The persistence of these challenges is well documented. In Ecuador, healthcare reforms implemented between 2007 and 2017 expanded the primary care workforce and introduced free access to services. While these measures improved overall coverage, they did not fully eliminate disparities in access and utilization. Rural populations, Indigenous communities, and individuals with lower socioeconomic status continue to experience disproportionate barriers to care [2]. Key differences in access to healthcare and perceptions of humanized care across population groups are summarized in Table 2, illustrating the gap between system-level expansion and equitable health outcomes. Evidence from regional and rural nursing studies indicates that addressing these inequities requires approaches grounded in relational engagement, the promotion of autonomy, and experienced comfort [1,36]. From a phenomenological perspective, humanized care is understood as an ethical and culturally embedded practice that acknowledges suffering, fosters trust, and restores dignity through meaningful relationships [5,11]. As such, it represents not merely an enhancement of care but a moral and practical necessity in addressing structural violence and advancing health equity.

Within this regional framework, Ecuador offers a particularly relevant context for the application of humanized community nursing. Ongoing efforts to integrate biomedical systems with Indigenous health traditions through intercultural health policies aim to harmonize diverse knowledge systems. However, these efforts remain incomplete, with Indigenous perspectives often underrepresented in formal healthcare delivery [29]. At the same time, structural inequities in resource distribution and service access persist. Indicators reflecting both progress and ongoing gaps in the Ecuadorian healthcare system are presented in Table 3, highlighting the complexity of translating policy into equitable practice. This convergence of reform, inequality, and intercultural interaction positions Ecuador as a valuable setting for operationalizing and evaluating integrated care models such as the RCC framework.

Community nursing in Ecuador is characterized by three interrelated features that align closely with the principles of humanized care. First, primary healthcare delivery emphasizes prevention and territorial presence, with nurses conducting home visits and engaging with families and communities rather than focusing solely on individual patients [1]. Second, intercultural health approaches require integrating Indigenous knowledge and practices alongside biomedical care, demanding cultural competence and adaptability from healthcare providers [24,29]. Third, community-based initiatives increasingly address social determinants of health by mobilizing family networks, community leaders, and outreach strategies to improve access to education, services, and preventive care [2,27]. These characteristics create a practice environment in which relational, participatory, and context-sensitive care is both necessary and operationally relevant.

The RCC framework provides a structured approach that aligns with these contextual realities. Its sequential organization reflects the natural progression of care in community settings and offers a practical mechanism for integrating theory into practice [5]. The Relate phase emphasizes establishing trust through culturally responsive engagement. In Ecuadorian contexts, this includes recognizing linguistic diversity, such as the use of Kichwa, acknowledging spiritual beliefs, and fostering open dialogue through empathetic listening and authentic presence. Such practices enable nurses to transform potential cultural barriers into opportunities for trust-building and meaningful connection [24,29].

The Capacity phase builds on this relational foundation by strengthening self-care abilities within the family and community context. Nurses assess existing capacities, provide tailored education, and integrate biomedical and traditional knowledge systems to support disease prevention and management. This approach enhances self-efficacy, promotes long-term adherence, and supports sustainable health outcomes, particularly in resource-limited environments [1,15,16,17]. By reinforcing autonomy, this phase reduces dependency on formal healthcare systems while aligning with broader public health objectives.

The Comfort phase focuses on achieving experienced comfort across the physical, psychospiritual, sociocultural, and environmental domains. In practice, this involves addressing living conditions, mitigating emotional distress associated with poverty and isolation, and evaluating care outcomes based on individuals’ lived experiences of dignity and well-being rather than solely on clinical indicators [3,4,19,20,21,35]. This outcome-oriented perspective is particularly significant for vulnerable populations, where perceived comfort directly influences satisfaction, adherence, and sustained engagement with care.

The sequential progression of Relate > Capacity > Comfort reflects the lived dynamics of community nursing in Ecuador, where trust is a prerequisite for empowerment and empowerment is necessary for achieving lasting well-being [1,5]. By aligning theoretical constructs with practice realities, the RCC framework provides a coherent, adaptable model for delivering humanized care in intercultural, resource-constrained settings.

Emerging evidence supports the relevance of this approach across Latin America. Studies indicate that humanized care is associated with improved treatment adherence, increased patient satisfaction, and strengthened community trust [3,5,23]. In Ecuador, nursing education initiatives that emphasize humanized care have demonstrated measurable improvements in cultural competence and ethical practice, reinforcing their alignment with national primary healthcare priorities [24,25,30]. Furthermore, the integration of community health workers highlights the effectiveness of relational, capacity-building, and comfort-oriented approaches in promoting equitable health outcomes [27].

Overall, the RCC framework offers a contextually grounded and scalable model for advancing humanized care in Latin America. By bridging theory and practice, it enables community nurses to navigate structural constraints while promoting dignity, autonomy, and well-being. In doing so, it reinforces humanized care as both an ethical obligation and a practical strategy for achieving meaningful, sustained health equity in diverse, resource-limited settings.

## 6. Competency Implications for Nurses: The RCC Framework in Professional Identity

The RCC framework conceptualizes professional competence in humanized community nursing as the integration of relational, self-care facilitation, and comfort-oriented capabilities rather than the accumulation of theoretical knowledge alone. Competence within this framework refers to the nurse’s ability to translate these capabilities into context-sensitive practice while recognizing the social, cultural, and organizational conditions that shape care delivery [1,5]. This distinction is important because humanized care cannot be achieved through individual professional attributes alone; its enactment depends on the interaction between nurse competencies, patient and community participation, and the structures and processes of the health system. The framework organizes these competencies into three mutually reinforcing domains. Relate concerns the establishment of therapeutic and culturally responsive relationships. Capacity concerns the assessment and strengthening of individuals’ and communities’ ability to participate in self-care, and Comfort concerns the assessment and improvement of physical, psychospiritual, sociocultural, and environmental well-being. Rather than representing rigid stages, the three domains describe interconnected functions that may occur simultaneously and recur throughout the care process. This structure reduces conceptual overlap while providing a practical pathway from interpersonal engagement to capacity-building and, ultimately, to patient-defined outcomes.

**Relate: Establishing the conditions for engagement**

The Relate domain translates Watson’s relational orientation into observable community nursing practices, including active listening, culturally responsive communication, shared decision-making, continuity of contact, and recognition of patients’ and families’ explanatory models of health [5,11]. In practice, these competencies may be expressed through allowing sufficient time for patients to describe concerns in their own terms, involving family members when culturally appropriate, using interpreters or meaningful communication strategies, and acknowledging traditional health practices before negotiating a care plan. Such practices are particularly relevant where historical inequities or institutional distrust may affect willingness to engage with formal health services [1,24,29]. The significance of Relate, therefore, extends beyond interpersonal communication. Trust can influence whether individuals disclose health concerns, accept recommendations, return for follow-up, and participate in preventive activities [3,28]. However, relational competence should not be interpreted as an individual responsibility to overcome structural distrust. Continuity of staffing, reasonable workloads, adequate consultation time, and organizational support are necessary conditions for nurses to establish sustained relationships. Thus, the framework positions relational competence as a professional capability that must be enabled by appropriate service conditions rather than as a substitute for them.

**Capacity: Supporting autonomy without transferring responsibility to the patient**

The Capacity domain operationalizes Orem’s emphasis on self-care by focusing on assessment of self-care ability, individualized education, shared goal-setting, and progressive transfer of responsibility according to patients’ capabilities and circumstances [13,14,15,16,17,32]. Practical applications may include identifying barriers to medication management, adapting health education to literacy and language needs, involving family or community networks where appropriate, and connecting individuals with available social or health resources. The objective is not to maximize patient independence irrespective of circumstances, but to strengthen feasible forms of self-management while recognizing when professional or family support remains necessary. Evidence from Orem-informed interventions indicates improvements in self-efficacy, quality of life, and health-related behaviors in several chronic and vulnerable populations [13,14,15,16,17,32]. Nevertheless, these findings should not be interpreted as direct validation of the RCC framework. They provide empirical support for one theoretical component incorporated into RCC. The effectiveness of capacity-building is also likely to depend on health literacy, socioeconomic resources, continuity of care, family support, and access to medicines and other essential services. Consequently, nurses cannot be expected to generate autonomy where structural conditions systematically restrict patients’ available choices.

**Comfort: Translating care into patient-relevant outcomes**

The Comfort domain incorporates Kolcaba’s proposition that nursing outcomes should include patients’ perceived physical, psychospiritual, sociocultural, and environmental well-being [19,20,22,23]. Its practical value lies in broadening outcome assessment beyond biomedical indicators. Nurses may therefore assess symptom relief alongside emotional distress, perceived dignity, safety within the home environment, social connectedness, and the extent to which care is culturally acceptable. Such an assessment can guide modifications to care plans and help identify social or environmental barriers not captured by conventional clinical measures. Existing evidence associates comfort-oriented approaches with patient satisfaction, perceived quality of care, adherence, and other experiential outcomes [19,20,23]. However, comfort is inherently influenced by individual expectations and sociocultural context, making standardized assessment challenging across communities. The RCC framework, therefore, treats comfort as a patient- and context-sensitive outcome rather than a uniform endpoint. This distinction is particularly important in intercultural care, where professional definitions of well-being may differ from those of patients, families, and communities [29].

**From individual competence to system-enabled practice**

A central implication of the RCC framework is that competencies cannot be implemented independently of the community healthcare system. Humanized care requires organizational and structural conditions that enable nurses to exercise relational, educational, and evaluative competencies. Relevant changes include adequate staffing and time for community encounters, continuity of care, access to referral and multidisciplinary services, culturally appropriate training, mechanisms for community participation, and collaboration with community health workers [22,27,39,40]. Educational curricula can support these competencies through supervised community placements, reflective practice, intercultural training, and integration of theoretical knowledge with real-world problem-solving [24,25,30,33,34]. This system’s perspective is particularly important in resource-constrained settings. Geographic isolation, workforce shortages, fragmented referral pathways, inadequate infrastructure, and competing clinical demands may restrict the feasibility of sustained relational engagement and individualized capacity-building [1,2,27,40]. Intercultural care introduces additional challenges involving language, traditional knowledge, differing explanatory models of illness, and unequal power relationships between biomedical institutions and local communities [26,29]. Community health workers may facilitate communication and cultural bridges, but their contribution also requires institutional recognition, appropriate training, and clear mechanisms for collaboration [27]. Accordingly, RCC should not be interpreted as assigning nurses sole responsibility for achieving humanized care. Rather, nurses function within a system whose organizational design can either enable or constrain the competencies described by the framework.

**Evidence boundaries and conceptual limitations**

The RCC framework currently represents a conceptual synthesis of established nursing theories and empirical literature rather than an empirically validated intervention model. Evidence supporting Watson’s relational principles, Orem-based self-care interventions, and Kolcaba’s comfort-oriented outcomes provides theoretical and indirect empirical justification for the three domains [5,11,13,14,15,16,17,19,20,23,32]. However, these findings cannot establish that their specific integration within RCC produces superior outcomes compared with existing community nursing approaches. Similarly, reported improvements in adherence, satisfaction, self-efficacy, or trust in studies of humanized or theory-informed care should be regarded as supporting evidence for individual components rather than evidence of RCC effectiveness [3,5,23]. The framework also has several conceptual limitations. First, its three domains may overlap in practice because therapeutic relationships can influence self-care capacity, while comfort may simultaneously facilitate trust and engagement. Second, the proposed progression from Relate to Capacity to Comfort should not be interpreted as a strictly linear causal sequence. Community care is iterative, and changes in comfort, trust, or self-care capacity may alter subsequent nurse-patient interactions. Third, the framework has not yet been tested across diverse community populations or health-system configurations, limiting the conclusions regarding its transferability. Fourth, operationalizing constructs such as authentic presence, cultural responsiveness, autonomy, and perceived comfort requires reliable indicators that can capture both measurable outcomes and context-specific experiences. Finally, a limitation of this conceptual review is the relatively limited availability of independent empirical research specifically addressing humanized community nursing in Ecuador. Consequently, some context-specific evidence is derived from a small number of author groups, including the authors’ previous work. Although the revised review incorporates broader independent evidence from Latin American and international contexts, the RCC Framework should therefore be interpreted as a contextually informed conceptual proposition rather than a framework with established generalizability to Ecuador or other settings. Further independent empirical studies are needed to evaluate and refine its applicability across diverse community nursing contexts.

**Figure 3:**
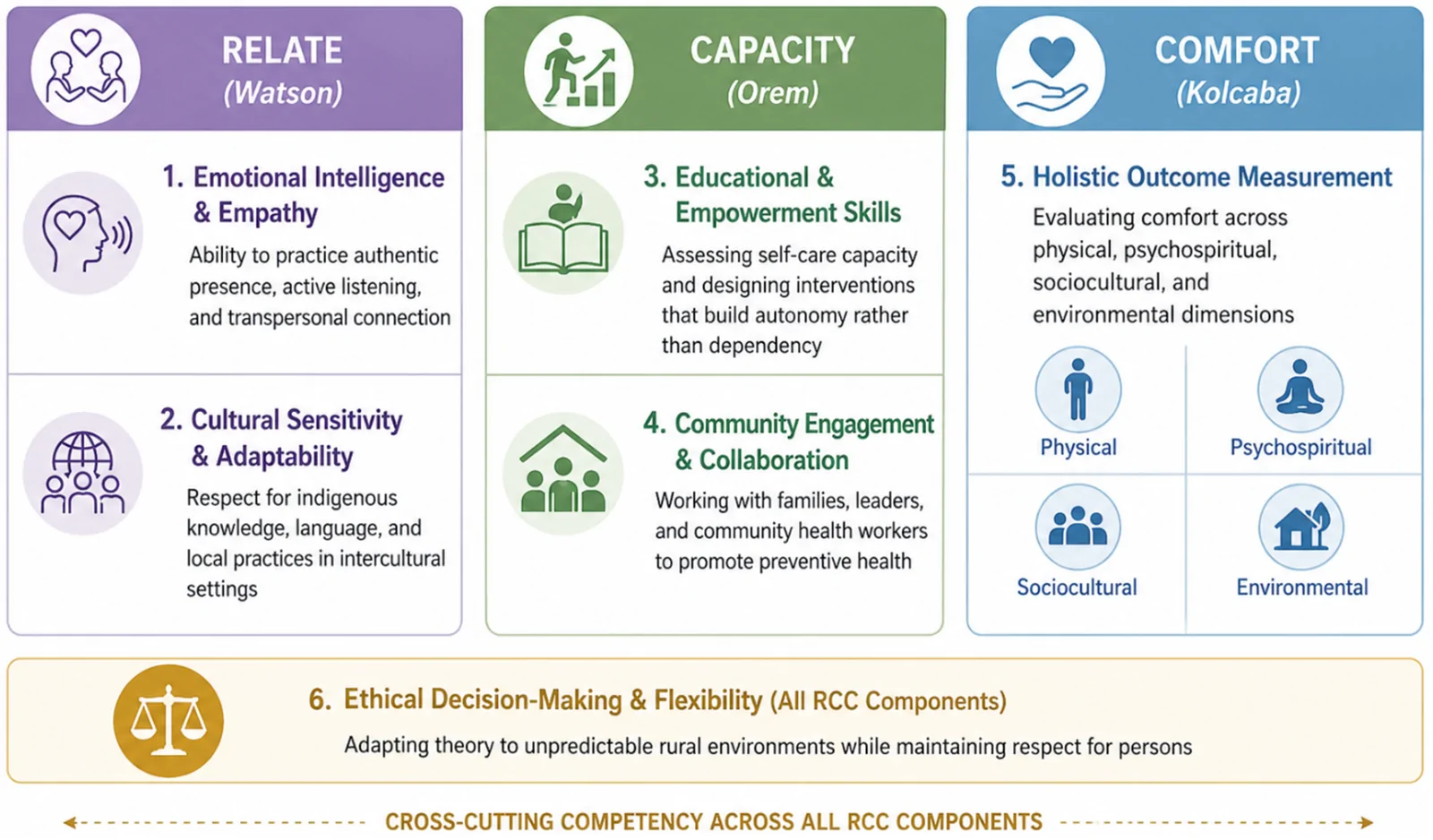
Competency framework aligned with RCC theories.

Accordingly, the principal contribution of RCC at this stage is conceptual, providing an integrative structure that connects relational practice, self-care facilitation, and patient-defined well-being within community nursing. Its empirical value remains to be established through prospective implementation studies, mixed-methods evaluation, and comparative research across different community and health-system contexts. Such studies should examine not only patient outcomes but also implementation processes, organizational readiness, nurse workload, community participation, cultural acceptability, and resource requirements. This approach would allow the framework to be evaluated as a system-level model of humanized care rather than as a set of competencies expected to be implemented by individual nurses alone (Table 4 and Figure 3).

## 7. Conclusions and Future Direction

Humanized care in community nursing across Latin America cannot remain a theoretical aspiration. It must be translated into structured, context-responsive practice. This review has addressed this need by proposing the RCC framework, an integrated model that operationalizes three foundational nursing theories, such as Jean Watson’s Theory of Human Caring, Dorothea Orem’s Self-Care Deficit Nursing Theory, and Katharine Kolcaba’s Comfort Theory, into a coherent, sequential approach. The framework reflects the lived progression of community nursing, beginning with relational engagement (Relate), advancing through the strengthening of self-care capacity (Capacity), and culminating in the achievement of experienced comfort (Comfort).

In doing so, the RCC framework addresses a critical gap in the literature, in which these theories have traditionally been applied in isolation and insufficiently aligned with the realities of community-based care in Latin America. Its contribution lies in three interconnected advances: the integration of relational engagement, self-care capacity, and experiential comfort into a unified conceptual core; the articulation of a sequential logic that mirrors real-world care processes; and its grounding in intercultural and primary healthcare priorities, particularly within the Ecuadorian context. Together, these elements transform theory into an actionable framework that is both conceptually rigorous and practically relevant.

The implications for nursing practice are significant. By providing a clear and adaptable structure, the RCC framework enables community nurses to move beyond task-oriented care toward embodied, humanized practice. It strengthens relational trust, supports patient autonomy, and prioritizes lived experiences of well-being, all of which are essential to improving adherence, satisfaction, and equity in resource-constrained, culturally diverse settings. In this sense, humanized care is repositioned not as an optional enhancement but as a strategic response to persistent social and health inequities across the region.

Looking forward, the advancement of the RCC framework depends on its empirical validation and contextual adaptation. Future research should focus on implementing the model across diverse community settings in Ecuador and Latin America, with particular attention to measurable outcomes such as trust-building, self-care efficacy, patient satisfaction, treatment adherence, and reductions in health disparities. Longitudinal and mixed-methods studies would be especially valuable in capturing both the experiential comfort and outcome-based dimensions of care. Such evidence will be essential for refining the framework and ensuring its scalability across different healthcare systems and cultural contexts.

Equally important are the implications for education and policy. Nursing curricula should integrate the RCC framework through experiential and community-based learning, enabling students to internalize relational, cultural, and ethical competencies alongside clinical skills. At the practice level, healthcare institutions should support environments that allow for continuity of care, community engagement, and culturally responsive interventions. From a policy perspective, strengthening primary healthcare systems, investing in intercultural training, and formally integrating community health workers are critical steps toward embedding RCC competencies within broader health strategies.

Ultimately, the RCC framework demonstrates that translating theory into practice is both achievable and necessary. By simplifying and sequencing complex theoretical constructs into a coherent model grounded in the realities of Latin America, it offers a pathway for transforming community nursing into a more equitable, relational, and dignity-centered practice. Humanized care, therefore, is not an abstract ideal nor a contextual luxury; it is an essential and actionable approach that can reshape healthcare delivery and outcomes for individuals, families, and communities across the region.

## Figures and Tables

**Table 1:** Theoretical Foundations of Humanized Care in Community Nursing.

Theory/Author	Core Concept	Key Elements	Application in Community Nursing	Contribution to Humanized Care	References
**Jean Watson**	Caring relationship	10 Caritas Processes (authentic presence, compassion, transpersonal relationships, empathy, mindfulness)	Establishing genuine relationships during home visits, intentional presence, and valuing emotional and spiritual dimensions	Establishes trust as the prerequisite for care acceptance in intercultural and rural settings	[5,6,9]
**Dorothea Orem**	Self-Care Deficit	Self-care agency, therapeutic self-care demand, supportive-educative nursing systems	Assessing family self-care capacity, designing empowering education, and promoting preventive practices within local beliefs.	Positions patients as active agents; nursing competence includes knowing when to support, teach, or step back to avoid dependency	[14,15,16,17]
**Katharine Kolcaba**	Comfort	Relief, ease, and transcendence in four contexts (physical, psychospiritual, sociocultural, environmental)	Creating safe home environments; addressing social distress linked to poverty and isolation; measuring success through lived experience	Shifts focus from clinical indicators to patient-perceived experienced comfort and satisfaction.	[3,19,20,21]

**Table 2:** Selected Health Equity and Humanized Care Indicators Relevant to Community Nursing in Ecuador and Comparable Latin American Contexts [2,11,27,28,36].

Indicator	Overall/Urban Level	Rural, Indigenous and Low-SES Groups	Key Trend/Disparity (2006–2023)	Implications for Community Nursing
**Unmet healthcare needs**	18% (2014)	Substantially higher	Decreased from 27% (2006), but relative inequalities persist	Demands relational trust-building and intercultural approaches
**Reduction in unmet needs**	Significant absolute decline	Relative gaps remain pronounced	Absolute improvements observed, yet disparities endure	Highlights the limits of reform without humanized practice
**Favorable perception of humanized care (post-COVID)**	37.7%	20.4%	Marked urban-rural gap	Lower rural relational engagement undermines care acceptance
**Unfavorable perception of humanized care**	25.6%	38.2%	Rural patients report significantly lower trust and presence	Requires priority focus on empathy and authentic dialogue
**Community health worker integration in primary care**	Moderately integrated	Under-integrated across six countries	Key cultural bridge, but structurally weak	Strategic partnership opportunity for capacity-building

**Table 3:** Healthcare Access, Equity, and Intercultural Challenges in Ecuador (2006–2025) [2,38].

Indicator	2006 (Pre-Reform)	2014 (Post-Reform)	Rural, Indigenous and Low-SES Groups	Persistent Challenge/Implication for Humanized Care
**Unmet healthcare needs**	27%	18%	Substantially higher than the national average	Relative inequalities endure despite absolute gains
**Primary care team coverage (EBAS)**	Limited	Significantly expanded	Lower coverage and utilization in remote areas	Geographic isolation limits equitable reach
**Socioeconomic disparities in service utilization**	High	Moderately reduced (absolute)	Relative disparities remain pronounced	Reforms are insufficient without relational and cultural adaptation
**Intercultural health integration**	Nascent	Asymmetrical and incomplete	Limited formal incorporation of indigenous systems	Cultural incompatibility hinders trust and effectiveness
**Workforce distribution (compulsory rural service impact)**	Uneven	Persistent shortages	Acute resource and cultural gaps in remote communities	Nurses require intercultural competence and relational skills

**Table 4:** RCC framework in Ecuador.

RCC Phase	Theoretical Foundation	Core Concepts	Key Nursing Actions in Community Settings	Expected Outcomes	Ecuador-Specific Practical Application	References
**Relate**	Jean Watson’s Theory of Human Caring	Authentic presence, transpersonal relationships, empathy, Caritas Processes, cultural respect	Active listening; sincere home visits; acknowledgment of cultural identity, indigenous languages (e.g., Kichwa), and spiritual dimensions; deliberate presence	Establishment of trust; care acceptance; stronger nurse–community bonds	Respecting indigenous greetings and ancestral knowledge during home visits, transforming cultural differences into genuine relational opportunities	[5,24,29]
**Capacity**	Dorothea Orem’s Self-Care Deficit Nursing Theory	Self-care agency, supportive-educative systems, empowerment, autonomy-building	Systematic assessment of family self-care capacity; design of empowering education programs; integration of ancestral knowledge with biomedical prevention strategies	Improved self-management; reduced self-care deficits; greater patient/family autonomy and self-efficacy	Delivery of chronic disease education in rural health brigades; promotion of preventive practices that respect local beliefs and eliminate dependency	[1,15,17]
**Comfort**	Katharine Kolcaba’s Comfort Theory	Relief, ease, and transcendence across physical, psychospiritual, sociocultural, and environmental contexts	Holistic response to social determinants (poverty, isolation); creation of supportive home environments; evaluation of comfort through patients’ lived experience	Enhanced holistic comfort; higher patient satisfaction; improved treatment adherence and quality of life	Family centered interventions that address emotional distress from rural isolation and structural inequalities; integration of traditional healing alongside biomedical care	[3,20,23]

## Data Availability

The original contributions presented in this study are included in the article. Further inquiries can be directed to the corresponding author.

## References

[1] López-Andrade C.I., Fuentes K.I.M. (2025). Enfermería rural en Latinoamérica: Cuidando en la adversidad en los últimos 10 años. Revisión sistemática. Enfermería Cuid. Humaniz..

[2] Quizhpe E., Teran E., Pulkki-Brännström A.M., San Sebastián M. (2022). Social inequalities in healthcare utilization during Ecuadorian healthcare reform (2007–2017): A before-and-after cross-sectional study. BMC Public Health.

[3] Espinel-Jara V.M., Tapia-Paguay M.X., Tito-Pineda A.P., López-Aguilar E.C., Fernández-Cusimamani E. (2025). Humanized and community-based nursing for geriatric care: Impact, clinical contributions, and implementation barriers. Nurs. Rep..

[4] Olvera J.C., Poma N.D.F., Castro K.M.Y., Encarnación J.J.A. (2025). Humanized care of the elderly in critical services: Ethical dilemmas, impact and intervention strategies. Salud Integral Comunitaria.

[5] Meneses-La-Riva M.E., Fernández-Bedoya V.H., Suyo-Vega J.A., Ocupa-Cabrera H.G., Paredes-Díaz S.E. (2025). Humanized care in nursing practice: A phenomenological study of professional experiences in a public hospital. Int. J. Environ. Res. Public Health.

[6] Afonso S.D.R., Padilha M.I., Neves V.R., Elizondo N.R., Vieira R.Q. (2024). Critical analysis of the scientific production on Jean Watson’s theory of human care. Rev. Bras. Enferm..

[7] Braz Evangelista C., Limeira Lopes M.E., Lima da Nóbrega M.M., Ferreira de Vasconcelos M., Gomes Viana A.C. (2020). An analysis of Jean Watson’s theory according to Chinn and Kramer’s model. Rev. Enferm. Ref..

[8] Teófilo T.J.S., Gomes Viana A.C., Oliveira J.D.S., Abreu W.J.C.P.D. (2026). Empathy in Jean Watson’s theory of human caring: Philosophical reflections on the Caritas^®^ processes and mindfulness. J. Holist. Nurs..

[9] Espinoza Padilla D.J., Guerra Guerrero V.T., Poblete Troncoso M. (2024). Projections of disciplinary nursing care from the philosophy of Jean Watson: A systematic review. Enferm. Cuid. Humaniz..

[10] Sadat Hoseini A.S., Beigzadeh A., Delzendeh M. (2025). Application of Jean Watson’s theory in nursing practice: A quasi-experimental study. J. Kerman Univ. Med. Sci..

[11] Meneses-La-Riva M.E., Suyo-Vega J.A., Fernández-Bedoya V.H. (2021). Humanized care from the nurse–patient perspective in a hospital setting: A systematic review of experiences disclosed in Spanish and Portuguese scientific articles. Front. Public Health.

[12] Watson Caring Science Institute Affiliates (2025). First Latin American Academic Affiliate—Universidad Santo Tomás, Chile (Watson Theory Application). https://www.watsoncaringscience.org/.

[13] Muniz V.D.O., Braga L.D.C.A., Araujo P.O.D., Santana P.P.C., Pereira G.S., Sousa A.R.D., Pedreira L.C., Carvalho E.S.D.S. (2022). Self-care deficit among older men in the COVID-19 pandemic: Implications for nursing. Rev. Bras. Enferm..

[14] Kim J.E., Dee V. (2024). Utilization of Orem’s self-care deficit nursing theory in self-care among older Korean immigrants with diabetes in the United States. Res. Theory Nurs. Pract..

[15] Khademian Z., Ara F.K., Gholamzadeh S. (2020). The effect of self-care education based on Orem’s nursing theory on quality of life and self-efficacy in patients with hypertension: A quasi-experimental study. Int. J. Community Based Nurs. Midwifery.

[16] Gonçalves M.F.A., Gonçalves M.F.I.R., Zerquera J.R., Diogo M.L., Valentim E.E. (2026). Community self-care, prevention practices, and treatment-seeking behaviors related to malaria: A qualitative study grounded in Orem’s self-care deficit theory. Front. Trop. Dis..

[17] Mercado N.D.C., Eraso C.O.N., Bastidas T.C.R., Espinoza Y.A.Y. (2025). Basic conditions and self-care requirements (Dorothea Orem) for breast cancer prevention in women in a municipality of Nariño, Colombia. Rev. Criterios.

[18] Westergren A. (2025). Commentary: Cultural adaptation and content validation of the Watson Caritas patient instrument for a Latin American Spanish context. J. Res. Nurs..

[19] Lin Y., Zhou Y., Chen C. (2023). Interventions and practices using comfort theory of Kolcaba to promote adults’ comfort: An evidence and gap map protocol of international effectiveness studies. Syst. Rev..

[20] Lin Y., Zhou Y., Chen C., Yan C., Gu J. (2024). Application of Kolcaba’s comfort theory in healthcare promoting adults’ comfort: A scoping review. BMJ Open.

[21] Aloustani S., Parsai M., Siyasari A.R., Sharifi Rizi M., Papi S., Zafarramazanian F. (2026). Applications of Kolcaba’s comfort theory to improve nursing practice: A narrative review. J. Nurs. Rep. Clin. Pract..

[22] Rachter L., Stampini M., Tomé R., Duryea S., Vinacur T. (2024). Care Across the Life Cycle: Building Care Systems in Latin America and the Caribbean. https://www.scribd.com/document/1075232838/Care-Across-the-Life-Cycle-Building-Care-Systems-in-Latin-America-and-the-Caribbean.

[23] Espinoza J.B.R., Talavera B.E.M., Medinal D.M., Michua M.D.L.Á.P. (2025). Effectiveness of the comprehensive-humanized care model in nursing consultation: A case study with older adults. Rev. Bras. Enferm..

[24] Cabrera P.R., Solorzano S.M., Enríquez J.M., Chávez-Arizala J.F. (2025). Humanized care of nursing students of the Adventist Technological Institute of Ecuador, Santo Domingo de los Tsáchilas. Community Intercult. Dialogue.

[25] Dávila M.F.V., Orellana C.F.V., Balseca S.L.A., Rojas Y.E.U. (2025). Experiences of humanizing care in nursing students: A phenomenological study. Healthcare.

[26] Bernal H., Froman R. (1993). Influences on the cultural self-efficacy of community health nurses. J. Transcult. Nurs..

[27] Zuñiga R.O., Parra-García I., Gómez-Barrera L.A. (2024). Overview of the participation of community health workers in primary health care in six Latin American countries and a proposal for their integration into the health system: A qualitative study. Fam. Pract..

[28] Meneses-La-Riva M.E., Fernández-Bedoya V.H., Suyo-Vega J.A., Ocupa-Meneses G.D.D., Ocupa-Cabrera H.G. (2023). Differences in humanized care perceived by hospitalized patients from urban and rural areas during the COVID-19 pandemic: Evidence collected in Peru. J. Educ. Soc. Res..

[29] Herrera D., Hutchins F., Gaus D., Troya C. (2019). Intercultural health in Ecuador: An asymmetrical and incomplete project. Anthropol. Med..

[30] Ponce R.A.C., Sabando M.Y.S., Jácome M.Á.E., Chávez-Arizala J.F. (2025). Humanized care in nursing students: Review of concepts and background. SAP Community Intercult. Dialogue.

[31] Lv J., Su Y., Tang H., Jiang X., Chen X. (2025). Humanistic nursing care management strategies: From formulation to implementation. Front. Public Health.

[32] de Souza Gonçalves L.M., Glass B.D., Soares L. (2026). Pharmacists’ professional and service qualifications for the care of trans, travesti, and gender-diverse people in southern Brazil: A grounded theory study. Front. Med..

[33] Guerra-Marmolejo C., Morante-García W., Berthe-Kone O., Chica-Pérez A., Dobarrio-Sanz I., Granero-Molina J., Ortega-Avila A.B. (2024). Nursing students’ perceptions of learning through a service learning programme with older adults living in poverty in a high-income country: A descriptive qualitative study. Healthcare.

[34] Montebon R.L., Bachanicha R.M., Ocariza R.D.P. (2023). Transformative reflections of nursing students and alumni volunteers on the community extension services of the university. Int. J. Sci. Res..

[35] Castro M.C.F.D., Fuly P.D.S.C., Santos M.L.S.C.D., Chagas M.C. (2021). Total pain and comfort theory: Implications in the care to patients in oncology palliative care. Rev. Gaúcha Enferm..

[36] Meneses-La-Riva M.E., Fernández-Bedoya V.H., Suyo-Vega J.A., Ocupa-Cabrera H.G., Ocupa-Meneses G.D.D., Diaz S.E.P. (2023). Perception of humanised care in hospitalised patients in a public institution in post-pandemic Lima, Peru. Acad. J. Interdiscip. Stud..

[37] Cavender A., Albán M. (1998). Compulsory medical service in Ecuador: The physician’s perspective. Soc. Sci. Med..

[38] Sánchez del Hierro G., Remmen R., Verhoeven V., Van Royen P., Hendrickx K. (2014). Are recent graduates enough prepared to perform obstetric skills in their rural and compulsory year? A study from Ecuador. BMJ Open.

[39] León-Castro D.L., Cabanillas-Chávez M.T., Meneses-La-Riva M.E., Suyo-Vega J.A., da Costa Polonia A. (2023). Dynamics of the nursing professional’s spirituality and religiosity to address humanized care in healthcare settings: A systematic review of Latin American experiences. Acad. J. Interdiscip. Stud..

[40] Méndez Toledo J.R. (2025). Benefits, challenges and strategies in the implementation of humanized nursing care in hospitalization: A narrative review. Enferm. Cuid. Humaniz..

